# Twenty years of the methadone treatment protocol in Ireland: reflections on the role of general practice

**DOI:** 10.1186/s12954-018-0272-4

**Published:** 2019-01-17

**Authors:** Ide Delargy, Des Crowley, Marie Claire Van Hout

**Affiliations:** 1 Irish College of General Practitioners, Dublin, Ireland; 2 Public Health Institute, Liverpool John Moores University, Liverpool, UK

**Keywords:** Ireland, General practice, Methadone, Opioid dependence, Harm reduction

## Abstract

**Background:**

Opioid dependence, characterised by socio economic disadvantage and significant morbidity and mortality, remains a major public health problem in Ireland. Through the methadone treatment protocol (MTP), Irish general practice has been a leader in the introduction and expansion of Irish harm reduction services, including opioid substitution treatment (OST), needle and syringe programs (NSP) and naloxone provision. These services have been effective in engaging opiate users in treatment, reducing human deficiency virus (HIV) and hepatitis C virus (HCV) transmission and reducing-drug related morbidities. Challenges remain in relation to choice of substitution treatments, timely access to OST services, adequate coverage of NSP, naloxone provision and increasing drug-related deaths.

**Methods:**

A narrative review was conducted and designed to present a broad perspective on the Irish MTP and to describe its history and development in terms of clinical care, stakeholder views and changing trends.

**Results:**

Three themes emerged from the analysis; *The History of the Methadone Treatment Protocol*, *Service User and Provider Views* and *Challenges and Developments*. Despite the initial concern about methadone maintenance treatment (MMT) in Ireland, increased participation by Irish GPs in the treatment of opioid dependence is observed over the last two decades. There are now over 10,000 people on methadone treatment in Ireland, with 40% treated in general practice. The MTP provides structure, remuneration and guidance to GPs and is underpinned by training, ongoing education and a system of quality assurance provided by the Irish College of General Practice (ICGP). Challenges include the negative views of patients around how methadone services are delivered, the stigma associated with methadone treatment, the lack of choice around substitution medication, waiting lists for treatment in certain areas and rates of fatal overdose.

**Conclusion:**

Twenty years of the MTP has been the mainstay of harm reduction services in Ireland. It has provided a network of specially trained GPs who provide methadone to over 10,000 patients across Ireland within a structured framework of training, quality assurance and remuneration. With the ongoing commitment of Irish specialists in the field of addiction medicine, further improvements to support and treat patients can be made.

## Background

Opiate use in Ireland as elsewhere is characteristic of socioeconomic disadvantage, low educational attainment and restricted economic opportunity [1, 2]. The most recent capture recapture study of opiate users indicates that opiate use in Ireland is now entering a stabilised phase, with a small decrease in use compared to 2006 and the rate of use among young adults aged 15–24 years declining, and with a visible ageing cohort effect of those in the 35 to 64 years age group [3]. In 2003, 6667 individuals were registered on the Central Treatment List (CTL) out of 15,000 opiate users [4]. Kelly et al. [5] estimated that in 2006 there were between 18,136 and 23,576 opioid users resident in Ireland, with the highest rate of heroin use in Europe at just over 7 cases per 1000 population, and small increases in the older drug using population and in Irish females. National prevalence and treatment data indicated at the time that opiate use was no longer confined to the greater urban context in the capital [1, 5–8].

The overall number of cases treated for problem opiate use (mainly heroin) increased between 2006 and 2015 [9]. Most recent trends indicate a concerning shift toward greater levels of poly substance use [5, 6, 9] with related treatment demand (i.e. benzodiazepines) since 2007, dependence rates on over the counter and prescribed opiates such as codeine since 2008 [10], increase in drug related deaths [9] and the emergence of potent and potentially fatal synthetic fentanyls in 2018. Treatment data in 2013 reports that incidence of treated problem substance use among Travellers is three times that compared to the general population (523 per hundred versus 173 per 100,000) [11], with reports in 2017 indicating increased vulnerability of this ethnic minority to problem opiate use [9, 12]. In 2016, the Health Protection Surveillance Centre reported that the rate of infectious diseases (human immune deficiency virus, (HIV) and hepatitis C (HCV) is declining among people who inject drugs (PWID), despite an increase in 2015 due to a reported HIV outbreak [13].

In terms of pharmacological options to treat opiate dependence, substitution treatment using methadone is most common in Ireland, with buprenorphine-naloxone currently available on a limited basis [10]. The Irish model of care for delivery of methadone treatment acknowledges the central role of the specialist trained general practitioner (GP) in primary care [14]. The Irish College of General Practitioners (ICGP) has played a central role in developing the Methadone Treatment Protocol (MTP) which provided for the delivery of methadone treatment in primary care in the Irish context.

A network of prescribing GPs work closely with statutory (funded and operated by the Health Services Executive; HSE) and non-statutory (part funded by the HSE through a service level agreement) organisations to optimise methadone treatment delivery. The network includes specialist GPs working in HSE addiction clinics and Level 1 and Level 2 GPs working in primary care. Methadone treatment is commenced by specialist GPs in either addiction clinics or in a general practice setting (Level 2 GP). Level 2 trained doctors are qualified to initiate treatment in general practice, stabilise doses and provide ongoing maintenance treatment [15]. Level 1 trained doctors are encouraged to manage patients registered to their practice with patient stabilisation occurring in specialised clinics or with a Level 2 trained doctor. Once the patient is stabilised on methadone, referral to a Level 1 GPs working in the community for ongoing care is encouraged. All patients on methadone are listed on the confidential Central Treatment List (CTL) with each patient linked to one specific prescriber and a single dispensing site. Both Level 1 and Level 2 contracts attract additional remuneration for GPs caring for opioid dependent patients. Since 1998, greater prescribing of methadone in primary care has occurred, with the number of trained GPs rising steadily each year. There are currently (mid 2018) a total of 1718 trained Level 1 GPs and 188 Level 2 trained GPs on the ICGP database. We present here a narrative review of the history and development of the Irish MTP, 20 years since its initial inception.

## Methods

A narrative review was conducted and designed to present a broad perspective on the Irish MTP, and describe its history and development in terms of clinical care, stakeholder views and changing trends. A comprehensive search was conducted on the National Documentation Centre for Drugs, Health Research Board database, with no restriction on date range or types of records. Key search terms used were methadone treatment, general practice, opioid dependence and harm reduction combined with Ireland. Databases searched included PubMed, Science Direct, EMBASE, PsycINFO, Cochrane library and Medline. No limits were placed on dates. Follow-up search strategies included hand searching relevant national websites including the Health Service Executive (HSE), Irish Prison Service (IPS), Departments of Health and Justice (DOH and DJ), the Irish Penal Reform Trust (IPRT), Health Surveillance Protection Centre (HSPC) and EMCDDA. A hand search of reference lists from published peer-reviewed studies was also undertaken. References were managed by the citation manager Endnote®. National experts and authors of existing papers were contacted to identify possible sources of unpublished and grey literature. The research team reviewed the relevant literature and agreed on the structuring of the review under the following three themes: *The History of the Methadone Treatment Protocol, Service User and Provider Views,* and *Challenges and Developments*.

### The history of the methadone treatment protocol (MTP)

**Fig. 1:**
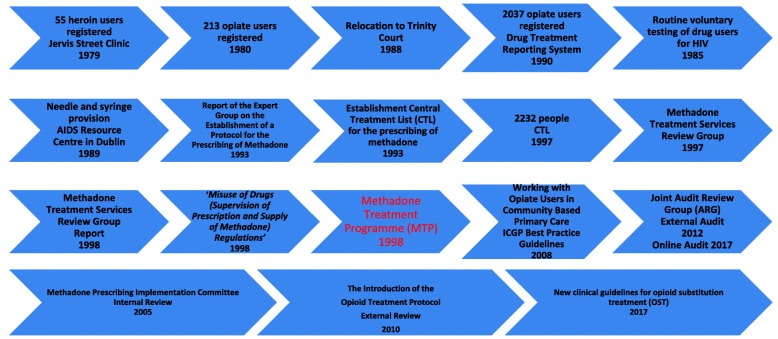
‘MTP Timeline’: the MTP key developments over time

The Drug Treatment and Advisory Service was established in Jervis Street hospital in 1969. In 1971, methadone was introduced as the standardised therapeutic approach for treatment of opiate dependence, with physeptone treatment available to a small cohort of patients attending the service. In 1979, the Jervis Street clinic in Dublin treated 55 heroin users; this figure rose to 213 in 1980 [16]. In the early 1980s Dublin experienced what can be described as ‘an opiate epidemic’ For a 20-year period, this was the only medical drug treatment facility in Dublin [17]. In 1988, following the closure of the Jervis Street hospital, the drug treatment clinic was relocated to a central Dublin site named Trinity Court. In 1990, the newly established Dublin Drug Treatment Reporting System [18] had 2037 opiate users treated in its expanding treatment system. Routine voluntary testing of drug users for HIV began in 1985 and over the first 2 years, 19% of those tested were diagnosed as being HIV positive. Needle and syringe provision was first provided in Ireland in 1989 in the former Eastern Health Board (EHB) AIDS Resource Centre in Dublin, in response to the heroin problem at the time [19]. Methadone services, mainly focused on detoxification, have been available since 1992 [20] and were initially restricted to the capital, Dublin [17]. The *‘Report of the Expert Group on the Establishment of a Protocol for the Prescribing of Methadone’* was conducted in 1993 [20]. It recommended that services should be developed using this protocol to make methadone available free of charge and national in coverage to all persons undergoing methadone treatment for opiate dependence. It also included recommendations on the type and concentration of methadone to be used, the roles of general practitioners and pharmacists, and the relationships between treatment centres, general practitioners and pharmacists. The CTL for the prescribing of methadone was established in 1993, and by June 1997, 2232 people were registered as on methadone treatment, see Fig. 1, MTP Timeline.

The Methadone Treatment Services Review Group was set up by the Department of Health and Children in 1997 to assess methadone use in the treatment of heroin dependence. In 1998, the report of the Methadone Treatment Services Review Group was published (Methadone Treatment Services Review Group, 1998) [21]. In October 1998, legislation was introduced as the *Misuse of Drugs (Supervision of Prescription and Supply of Methadone) Regulations* which stipulated the regulatory structures for treating opioid-dependent patients with methadone. It was also intended to encourage GPs to become involved in the treatment of drug dependence. The 1998 MTP was written to guide opioid-dependent treatment delivery in primary care, with detailed protocols for methadone prescribing, guidance and standards for patient management and care, specialist training requirements for GPs and protocols for clinical audit. As GPs were subsequently contractually required to have specific training if they wished to provide methadone treatment, the ICGP developed and delivered a number of training programmes to enable GPs to fulfil these contractual requirements: Level 1 GPs were provided with a daylong training programme while more experienced GPs, who had been working with opiate-dependent patients prior to the introduction of the protocol, were offered Level 2 status via a ‘grandfather clause’. Best practice guidelines, *Working with Opiate Users in Community Based Primary Care* were published by the ICGP [22] and were issued to all GPs participating in the MTP. Ongoing audit formed part of the contractual obligations and the ICGP convened an audit review committee in conjunction with the EHB with the purpose of developing an audit process for GPs participating in the MTP. This Joint Audit Review Group (ARG) [23] developed a model of external audit of adherence to the MTP, which was the first of its kind in Irish general practice. In addition to overseeing the audit process, the ARG was given the remit of overseeing training and continuing medical education (CME) for GPs participating in the MTP delivered by the ICGP. The audit process underwent a number of changes since then and, since 2017, utilises a blended method of online self-audit supplemented by random external audit [24]. The aim of this novel audit process is to assess the quality of care provided by GPs to patients on methadone maintenance treatment (MMT) so as to ensure that patient care meets national and international best practice standards. Furthermore, it aims to enhance practice-based learning and reflective practice in order to improve patient care and safety, minimise methadone diversion, reduce drug overdose rates, address associated health conditions and optimise patient rehabilitative outcomes within the practice [24].

**Fig. 2:**
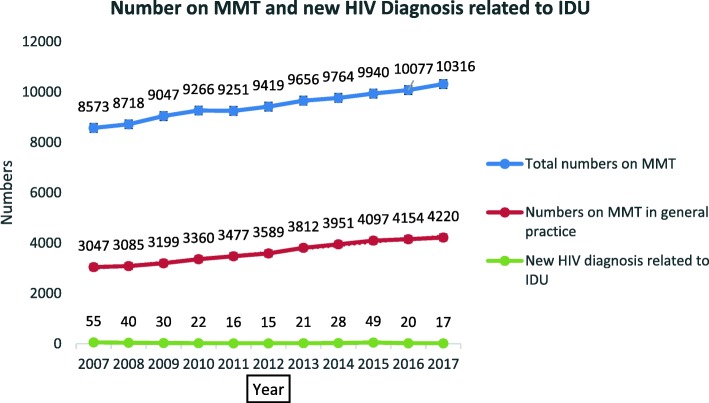
‘Number on MMT and new HIV Diagnosis related to IDU’: the numbers on MMT and new HIV diagnoses relating to IDU over time

Reviews of the MTP were conducted internally in 2005 by the *Methadone Prescribing Implementation Committee* itself and externally in 2010 [25]. The 2010 review was an external review of the MTP undertaken to inform and maximise treatment provision and assess clinical governance and audit, referral pathways, doctor enrolment, training (Levels 1 and 2) and GP coordination [25]. The review commented on improved prescribing and quality of independent practitioner practice and advised the need to maximise treatment provision and referral pathways. It also commented on a number of other issues, timely responses to requests for detoxification to be reviewed as part of a service audit process (see *National Drugs Rehabilitation Framework* Working Group on Drugs Rehabilitation) [26], rural service development, improved integration between and among services, improved clinical governance and audit, a need to review benzodiazepine prescribing (see *Report of the Benzodiazepine Committee* Department of Health and Children 2002) [27], changing urine analysis regimes, prescribing of methadone in police stations and the expansion of the number of Level 2 doctors with greater emphasis on transfer of patients from Level 2 to Level 1 doctors. Farrell and Barry [25] also commented on the inclusion of buprenorphine and naloxone treatment modalities and the need to revise the title to *The Opioid Treatment Protocol,* see Table 1 and Fig. 2.

**Table 1:** Number of patients on MMT by year, CTL 2007-2017

Year	2007	2008	2009	2010	2011	2012	2013	2014	2015	2016	2017
Total numbers on MMT	8537	8718	9047	9266	9251	9419	9655	9764	9940	10,087	10,316
Numbers of MMT with GPs	3047	3085	3199	3360	3477	3589	3812	3951	4097	4184	4220
New HIV cases related to IDU	55	40	30	22	16	17	21	28	49	20	17

### Service user and provider views

Early studies on Irish treatment provider experiences of methadone were mixed [28]. Service users described negative aspects centring on the patient lack of choice, humiliating experiences in consuming methadone in a public space, difficulties complying with punitive contracts and urine screening, and engaging with uncaring service providers [29–32]. Service user views were reported to centre on methadone as vital to initial stages of stabilisation, and that, due to the lack of alternatives to methadone, treatment regimens and patient experiences became restrictive and confining and seen as the only solution representative of the *one size fits all* phenomenon [32, 33]. A 2012 study highlighted the influence of the GP in supporting recovery [34]. Studies conducted in 2013 with GPs and service stakeholders [35, 36] and in 2016 indicated a generally positive attitude of prescribing GPs toward methadone treatment [14].

**Fig. 3:**
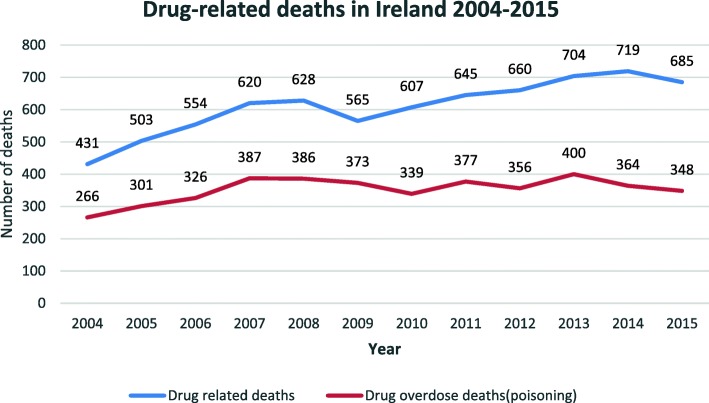
Three ‘Drug related deaths in Ireland 2004 to 2015’: the drug-related deaths in Ireland from 2004 to 2015

The outcomes of a national treatment and rehabilitation outcome study were reported in 2009 and showed high retention rates in treatment, high levels of completion of detoxification programs, significant reduction in all illicit drug use (heroin, cocaine, benzodiazepine, cannabis and illicit methadone), reduction in criminal activity, improved physical and health, reduction in risky drug taking behaviour and injecting drug use (IDU), improved uptake in training and employment and accommodation. Of note was that the incidence of non-fatal overdose remained constant [6, 37], see Table 2 and Fig. 3.

**Table 2:** Number of deaths, by year, NDRDI 2004 to 2015

Year	2004	2005	2006	2007	2008	2009	2010	2011	2012	2013	2014	2015
All deaths	431	503	554	620	628	656	607	645	660	704	719	695
Poisonings	266	301	326	387	386	373	339	377	356	400	364	348

(*N* = 7422)

### Challenges and developments

Complexities around methadone provision in Ireland in the initial years centred on negative attitudes toward drug users and differences of opinions around abstinence as an ultimate goal [38–40]. The MTP is restricted to the medico-pharmacological treatment of stabilised drug-dependent patients, with minor reference to psychotherapeutic treatment (Kenny and O’ Carroll) [41]. The absence of a framework for the use of psychotherapeutic interventions is deemed by Kenny and O’ Carroll [41] to warrant improvement.

A number of non-statutory agencies have a national brief and have in recent years advocated for the expansion of treatment, the decriminalisation of drug use and the setting up of drug consumption rooms. Many provide support and advocacy groups and a number of the larger agencies provide residential detoxification facilities [24]. A need for a widespread increase of detoxification services at both community and residential levels continues, along with a strengthened focus on family member support, dual diagnosis and mental health [26, 32, 42–51].

Regional treatment structures continue to struggle to meet the demand outside of the greater Dublin area, amid increasing drug-related deaths where methadone is implicated particularly within poly substance use, and changes in type of opioid abuse [24, 52]. Irish studies have reported on the elevated risk of drug-related mortality in methadone maintenance treatment during treatment initiation, transition, post drop out or discharge [53, 54]. Rates of benzodiazepine use implicated in over dose deaths are concerning for patients on methadone [54, 55]. Improved monitoring, risk assessment and methadone treatment retention strategies are needed to inform national drug overdose plans and overdose prevention [52, 56].

Given the increased trend in problem drug use among ethnic groups, current Irish opioid substitution guidelines are mainstream and do not refer specifically to the cultural considerations of ethnic minorities such as the Traveller community [57]. Given the complexities of methadone maintained patients in terms of multi morbidity and chronic illness, O’Toole et al. [58] recommended a more holistic approach to integrating MMT with general medical needs; the increased role of the GP Practice nurse; GP incentives to train in MMT, which any formal review of MMT GP remuneration should take into account the additional workload of taking care of MMT patients; and the promotion of better record-keeping with regard to chronic diseases and virology. Roy et al. [59] more recently have recommended an increased awareness of cardiac safety guidelines, including relevant clinical and family history, baseline, and trough dose ECG monitoring, should be incorporated into methadone maintenance therapy protocols.

Several additional key developments have occurred. Enhanced multi-sited data modelling will be utilised to track and monitor outcomes for policy makers, service providers and service users [60]. Van Hout and McElrath [61] have reported on the development of service user fora, designed to advocate and empower service users of MMT services in Ireland within the treatment care pathways and beyond. The National Poisons Information centre reported on 16 paediatric admissions for methadone toxicity in children under 4 years old in the period 2005–2014, and in response, a safe storage of methadone protocol has been rolled out nationally [62]. With regard to choice, at the time of writing in August 2018, buprenorphine-naloxone (Suboxone®) is prescribed to approximately 100 patients predominantly with codeine dependence or other opiate-based medications and with very little general practice involvement.

## Discussion

The MTP in Ireland represents a model of care which uses existing primary care resources for maximum benefit. As was the case in other jurisdictions, the drivers for devising the MTP in Ireland included the emergence of HIV among intravenous drug users and the unregulated prescribing of methadone by untrained GPs and pharmacists [63, 64]. The lack of co-ordination around the delivery of services prior to the introduction of the MTP contributed to poor practices, increased risk for patients and the refusal of GPs and others to look after drug users. Attitudes among health care professionals 20 years ago prior to the inception of the Irish MTP were that the opioid-dependent patient group were problematic, difficult to deal with and would create chaos in the surgery setting. Despite evidence to the contrary, there was also a large degree of scepticism among health care professionals about the benefits of methadone, particularly methadone maintenance as abstinence-based treatments and models of care were the norm at the time. The development of a new model of care in the form of the MTP represented recognition of harm reduction as a treatment option and provided a regulated structure for delivering MMT services. It also provided a legitimate *opt out* for GPs who did not wish to participate in the MTP; GPs could choose not to participate in the required addiction training and were consequently not eligible to provide MMT. The rationale for empowering GPs to become involved in treating drug users emerged from the UK and wider European experience [65, 66]. A national retention in treatment study also supported the view that the treatment of patients in primary care was a protective factor for retention in treatment [67].

Official endorsement for the MTP from the ICGP as the professional body for GPs was crucial in making it acceptable for GPs to get involved in this work. Prior to the introduction of the MTP, some of the messages emanating from the professional body were urging caution about the safety aspects of GP involvement in provision of MMT. The design of the protocol and regulatory controls around delivery of services gave GPs a greater sense of professional assurances and satisfaction in delivering services to drug users in their own practice [14]. GPs also played a key role in the delivery of services in specialist addiction centres with most of Health Services Executive (HSE) specialist services delivered by experienced Level 2 GPs. Current negotiations for a new general medical services (GMS) GP contract in Ireland include recommendations that all GPs should have completed the Level 1 training programme and be eligible to take a Level 1 contract. The ICGP continues to encourage all GP training schemes to avail of the Foundation Training in Substance Misuse (Level 1 training) which is available free of charge to all GP registrars and members of the ICGP.

The evidence from patients on the MTP suggests that it has contributed significantly to the improved health and wellbeing of many drug using patients [6]. One of the major achievements on the MTP over the past 20 years is the containment of HIV transmission among Irish people who inject drugs (PWID). At the time of the design and introduction of the MTP, there was major concern around rates of HIV infection and AIDS among small communities in the Dublin Inner City [68]. Over the past decade, the rates of new infections have been modest and reflect the impact of the overall harm reduction strategy which combined the provision of OST and the development and expansion of NSP. The number of new HIV diagnoses among injecting drug users in Ireland more than halved over the period from 2004 to 2009, and only 12 new infections were notified in 2017 among PWID [57] An unexpected increase in HIV notifications which occurred in 2015 were related to increased injecting use of new psychoactive substances (NSP) among homeless and chaotic PWID [69]. This highlights the need for vigilance and lack of complacency with regard to ongoing support of harm reduction services in Ireland.

The success with Hepatitis C infection has not been so marked, and there is a substantial sub population of current and former injectors who have chronic HCV-related liver disease [70–72]. The challenge of treating and managing such individuals with the new direct acting anti-retroviral (DAA) therapies will demand substantial medical resources including the transfer of HCV assessment and treatment into community addiction services and primary care [73, 74]. The need to scale up HCV treatment to levels where it provides for ‘treatment as prevention’ will be a major challenge over the next decade [57, 73–75]. The provision of MMT in the Irish Prison Service (IPS) was of further benefit to the introduction of the MTP. Until treatment became more widespread and continuity of care could be guaranteed on entry or exit from prison, opiate users who were serving sentences were offered detoxification only. This resulted in increased risk of HIV and hepatitis C transmission as well as the increased risk of fatal overdose on release. Policy within the IPS reflected the benefits identified with OST and prisoners are now either commenced on OST where appropriate or continued on their existing treatment regime when incarcerated. Re-engagement with their community-based OST treatment service is now an important risk management element of the prison discharge policy [76].

Despite the benefits of the MTP in Ireland over the past 20 years, significant challenges remain today. Access to treatment can be difficult specifically in areas outside of Dublin where waiting lists to access treatment continue to exist. It remains a concern that some PWIDs can wait long periods of time to access treatment which may reflect a rigidity within the existing protocol and how it is being interpreted in certain areas. The lack of choice in terms of which substitution medication is available is another constraint as buprenorphine-based medication is currently available on a limited basis only. Lack of flexibility with regard to geographical location of treatment is a factor which can become a barrier to treatment access. In some rural areas, patients may have long journeys to travel to access treatment and in areas where availability of public transport is an issue this can significantly influence both access and retention in treatment [24].

The single biggest challenge related to the MTP in Ireland today is the level of drug-related deaths [52]. The ICGP convened a working group to discuss strategies to minimise drug-related deaths as far back as 2007 [77]; however, drug overdose death rates have remained stubbornly high over the past decade with 224 people dying in 2015 [78]. Most of those who died were male and were in their late Thirties. The mean age of victims in 2015 was 39 years, the highest ever recorded, mainly due to the increase in deaths in those aged 55 years or older compared with 2014. The reason for this increase is not yet known, although more than half of deaths among those aged 55 years or older were among women. Opioids were most commonly associated with drug-induced deaths, although they were frequently found together with other psychoactive substances, such as alcohol and prescription medicines. Drug users not on OST are at greater risk from opioid- and methadone-related deaths. The drug-induced mortality rate among adults (aged 15–64 years) in Ireland was 70 deaths per million in 2015, which is more than three times the most recent European average of 21.8 deaths per million.

## Conclusion

Recent trends suggest a stabilisation or reduction in Irish opioid dependence rates yet opioid use remains a major public health problem. The MTP was put in place 20 years ago to enable the expansion of harm reduction services in Ireland in order to combat the high rates of HIV infections and the deaths associated with an increasing heroin epidemic in socially deprived areas of Dublin. Its introduction was controversial with mixed support from politicians, policy makers and medical professionals. The evidence-based harm reduction model challenged the existing traditional abstinence model of addiction treatment. The MTP is effective in supporting the retention of patients in MMT, reducing HIV and HCV transmission and improving the health and social functioning of opioid users. Despite the successes of the MTP, many challenges remain, including negative views of patients around how services are delivered, the stigma associated with methadone treatment, the lack of choice around substitution medication, waiting lists for treatment in certain areas and the rates of fatal overdose. The first 20 years has taken the Irish leaders in this field of medicine on a journey of improvement, advocacy and political lobbying in order to get the best possible services for patients in need of OST.

The ICGP has been a leader in the expansion of MMT services and has been instrumental in mainstreaming the care of this marginalised patient group in general practice. It has developed accessible training and has continuously made efforts to make substance misuse a mainstream issue in primary care rather than a niche area for a small number of GPs. It has been instrumental in developing a quality assurance audit with an educational focus at its core. This structured framework of training and quality assurance along with increased remuneration helped to allay the fears of many GPs who were reluctant to get involved in methadone treatment. Today, a network of specially trained GPs provides methadone to over 10,000 patients across Ireland. Reducing stigma and normalising OST in primary care is a challenge particularly in the context of ongoing criticisms of long-term maintenance treatment [79–81]. The MTP has provided a structure and support for GPs prepared to provide holistic care to drug users. As the range and type of substance misuse continues to evolve, GPs have a key role in identifying patients who may have a problem. Over the counter (OTC) codeine abuse as well as opiate analgesic dependency are a growing cause for concern and general practice is a less stigmatising environment to offer care [79]. Raising awareness and equipping GPs to meet the needs of their patients who may have substance use issues is of paramount importance if lives are to be saved. This area of medicine should no longer remain an *opt in* option; it is time to ensure that every GP is adequately trained to service their patients.
